# Establishment of an indirect ELISA method for detecting bovine coronavirus antibodies based on N protein

**DOI:** 10.3389/fvets.2025.1530870

**Published:** 2025-02-05

**Authors:** Qiang Liu, Xiaoxia Niu, Lingling Jiang, Gang Zhang, Pu Wang, Sinong Zhang, Weifeng Gao, Huichen Guo, Yujiong Wang, Yong Li

**Affiliations:** ^1^Key Lab of Ministry of Education for Protection and Utilization of Special Biological Resources in Western China, School of Life Sciences, Ningxia University, Yinchuan, China; ^2^State Key Laboratory of Veterinary Etiological Biology, College of Veterinary Medicine, Lanzhou University, Lanzhou Veterinary Research Institute, Chinese Academy of Agricultural Sciences, Lanzhou, China

**Keywords:** bovine coronavirus, N protein, eukaryotic expression, indirect ELISA, antibody detection

## Abstract

Bovine Coronavirus (BCoV) is a significant pathogen responsible for neonatal calf diarrhea, winter dysentery in adult cattle, and bovine respiratory diseases. Infection with the virus can result in hemorrhagic diarrhea, decreased milk production, and potentially fatal outcomes in cattle, leading to considerable economic repercussions for the cattle industry. Efficient management of BCoV relies on swift and precise detection techniques. CHO cells were utilized to express a secreted recombinant nucleocapsid protein (N), whereby rabbit polyclonal antibodies (pAb) were generated through immunization. An indirect enzyme-linked immunosorbent assay (iELISA) based on N protein was established for the detection of BCoV antibodies. Reaction conditions were optimized using a checkerboard approach, with the optimal antigen concentration at 1.25 μg/mL and the optimal antibody dilution at 1:200, the cutoff value distinguishing negative and positive serum samples was 0.986. The sensitivity test indicated that this rabbit pAb had a maximum dilution of 2^18^ within the assay range, did not cross-react with BHV-1, BVDV, BRV, and BRSV positive serum samples, and shown great specificity. The developed iELISA method and commercial kit were used to test 58 bovine serum samples, and the concordance rate was 94.83%. In summary, we have developed a cost-efficient and precise iELISA method based on N protein that serves as a useful diagnostic tool for BCoV in clinical samples and epidemiological research.

## 1 Introduction

BCoV is a pathogen responsible for inducing both gastrointestinal and respiratory illnesses in bovine populations. BCoV infections result in clinical manifestations including diarrhea, dehydration, fever, and respiratory distress in cattle. Furthermore, BCoV may lead to numerous clinical issues, impacting the overall health and productivity of cattle populations (1). The influence of BCoV presents a significant risk to herd health management and sustainable livestock husbandry. Although current vaccines mostly focus on calves, vaccinating adult cattle is essential for managing BCoV infections, decreasing viral transmission to calves, and alleviating enteric and respiratory diseases in all age groups within the herd. Meanwhile, BCoV is a zoonotic illness, with BCoV-like viruses identified in both wild ruminants and humans. The cross-species transmission of BCoV presents a significant challenge to public health safety between these groups (2). Timely and precise diagnosis is essential for effective preventative and control strategies. The current ELISA test kits for BCoV viruses suffer from the disadvantages of a long purchasing period and high price (3), necessitating the urgent establishment of a simple and sensitive assay to assess the current epidemiological status of BCoV and to furnish foundational data for the formulation of prevention and control strategies.

BCoV is a member of the *Betacoronavirus* genus in the *Coronaviridae* family. The genome measures around 31 kb and comprises 13 open reading frames (4). Among these, five principal structural proteins encode the hemagglutinin-esterase protein (HE), spike protein (S), membrane glycoprotein (M), envelope protein (E), and nucleocapsid protein (N). The N protein, a phosphorylated basic protein, is predominantly located within viral particles. It forms binds with the viral genomic RNA, preserving particle stability and integrity. The N protein elicits elevated levels of particular antibodies in the host due to its numerous antigenic epitopes. The N protein is highly conserved among BCoV strains and is expressed in abundance during viral replication, making it an ideal target for diagnostic reagent development.

To date, various molecular biology diagnostic methods have been developed for the detection of BCoV, including quantitative PCR (5), indirect immunofluorescence (6), loop-mediated isothermal amplification (7) and recombinase polymerase amplification (8). These methods are essential for detecting BCoV infections but are limited in their application. They require expensive and complex equipment, skilled technicians to operate, and have long experimental cycles, making them unsuitable for direct on-site testing. Conversely, ELISA is a rapid, convenient, and highly specific method for extensive disease sample analysis. It has benefits like precision, elevated sensitivity, user-friendliness, and excellent reproducibility, rendering it the preferred approach for clinical sample analysis in veterinary pathogen identification (9).

In this study, we constructed the pcDNA3.1-N recombinant expression plasmid and successfully expressed and purified a secreted recombinant N protein in CHO-K1 cells, immunized New Zealand Large White rabbits to obtain pAb, and established an iELISA based on the purified N protein for the detection of BCoV antibodies, aiming to improve diagnostic capabilities and epidemiological investigations.

## 2 Materials and methods

### 2.1 Plasmids, cell lines, animals, and viral strains

The plasmids pcDNA3.1(+) and pcDNA3.1-eGFP were sourced from GenScript Biotech Corporation (Nanjing, China). Chinese hamster ovary cells (CHO-K1) were purchased from the Cell Bank of the Chinese Academy of Sciences (Shanghai, China). These cells were cultured in a DMEM/F12 medium enriched with 10% fetal bovine serum to ensure optimal growth conditions. Male New Zealand Large White rabbits were acquired from Ningxia Medical University (Yinchuan, China), and bovine positive serum samples for BCoV, bovine diarrheal virus (BVDV), bovine rotavirus (BRV), bovine respiratory syncytial virus (BRSV), and bovine herpesvirus (BHV-1) were stored by our laboratory.

### 2.2 Construction of eukaryotic expression vector for recombinant N protein

The western and northern regions of China are deemed essential for the cattle business. Consequently, we downloaded the amino acid sequences of the N protein from seven strains of Bovine Coronavirus (BCoV) originating from Northwestern China using the NCBI database. Homology analysis revealed that the sequences were highly conserved, with homology greater than 97.9%. The transmembrane region and signal peptide sequence of the N protein were predicted using DeepTMHMM. N-glycosylation and O-glycosylation sites were forecasted with NetOGlyc and DictyOGlyc, respectively, while potential B cell epitopes were identified via the BepiPred website.

A signal peptide (MKWVTFISLLFLFASAY) was added to the N-terminus and a 6 × His tag to the C-terminus, accompanied by a Kozak motif (GCCACCATGG) preceding the start codon to enhance eukaryotic translation efficiency (10, 11). Restriction sites *Eco*RI and *Kpn*I were inserted at both ends of the gene, which was subsequently cloned into the pcDNA3.1(+) vector, named pcDNA3.1-N. The sequence was optimized and synthesized by GenScript using CHO cells as the host. The recombinant plasmid was transformed into DH5α cells and cultured overnight in LB liquid medium with 100 μg/mL ampicillin. The plasmid was isolated with an endotoxin-free plasmid mini-prep kit and validated through restriction digestion and DNA sequencing.

### 2.3 Expression and purification of recombinant N protein

A total of 1.2 × 10^6^ CHO-K1 cells were seeded onto a 6-well plate and incubated overnight. Once the cells reached approximately 90% confluency, the medium was replaced with fresh complete culture medium. Lipofectamine 3,000 transfection reagent was used to transiently transfect the cells with the recombinant expression plasmids pcDNA3.1-N and pcDNA3.1-eGFP. Following the established protocol (12), the culture medium was discarded after 24 h, and the cells were subsequently cultured in complete medium supplemented with 800 μg/mL G418. The growth of the CHO-K1 cells was monitored daily, and the medium was refreshed every 48 h to remove dead cells. Incubate for 3 days after the last media change, the cell supernatant was collected and purified using a gravity column filled with Ni-NTA agarose (IMAC) resin (Huiyan biotechnology, Wuhan, China). The purified N protein was then identified by Western blotting (WB) using a sheep anti-rabbit Anti-6 × His tag monoclonal antibody (Abcam, Shanghai, China).

### 2.4 Rabbit immunization and antiserum titer determination

Endotoxin-free recombinant N protein was mixed in a 1:1 ratio with Freund’s Complete Adjuvant (Sigma, Shanghai, China) and administered via multiple subcutaneous injections in the dorsal area of rabbits, with a negative control group established concurrently. Immunizations were carried out biweekly for a total of four times. Only the initial immunization utilized the complete adjuvant, while subsequent ones employed Freund’s Incomplete Adjuvant. After 50 days of immunization, the rabbits were deeply anesthetized, and cardiac puncture was performed to obtain antiserum. The titer of the post-immunization antiserum was subsequently assessed by indirect ELISA, employing the pure N protein as the coating antigen.

### 2.5 Purification and identification of rabbit antiserum

In this experiment, rabbit antiserum was purified utilizing gravity columns filled with Protein A Focurose HR resin, with all operations performed at 4°C to reduce protein degradation (13). Briefly, the antiserum was mixed with an equilibration buffer at a 1:2 ratio, filtered through a 0.22 μm filter, and then loaded onto the pre-equilibrated purification column. The column was allowed to stand for 1 h, followed by elution twice with 3 mL of Glycine buffer (pH 3.0), and each tube was neutralized with 80 μL of Tris–HCl buffer (pH 8.8). SDS-PAGE samples were prepared, and the results of the purification were analyzed using Coomassie Brilliant Blue staining. The purified pAb were used as primary antibodies to determine if they could specifically recognize the recombinant N protein via Western blot analysis.

### 2.6 Indirect immunofluorescence

The indirect immunofluorescence assay (IFA) was employed to detect the interaction between rabbit pAb and BCoV (14). MDBK cells in good growth condition were seeded at 1 × 10^6^ cells/well in a 6-well plate. Upon reaching approximately 80% confluency the next day, BCoV was added per well for incubation. After 48 h, cytopathic effects observed under a microscope indicated readiness for IFA. The purified rabbit pAb were diluted 1:2000 as the primary antibody, and TRITC–conjugated Goat Anti-Rabbit IgG (H + L) (Proteintech, Wuhan, China) was diluted 1:1000 as the secondary antibody for incubation. Fluorescence was then examined under a fluorescence microscope. Furthermore, an Anti-6 × His tag monoclonal antibody was employed as the primary antibody in an IFA to detect the expression of N protein in pcDNA3.1-N transfected CHO-K1 cells.

### 2.7 Optimization of iELISA conditions based on N protein

A checkerboard titration method was employed to ascertain the optimal coating concentration of the antigen and the dilution of the antibody. The recombinant N antigen was diluted in carbonate coating buffer to an initial concentration of 10 μg/mL, followed by serial two-fold dilutions. Three replicates were prepared for each dilution, with 100 μL added to each well of an ELISA plate, which was then incubated overnight at 4°C. The following day, the liquid was removed, and the plate was dried. Each well was treated with 150 μL of blocking agent, comprising 3% BSA, 5% BSA, and 5% skim milk powder (SMP), and incubated at 37°C for 2 h. Following three washes of the plate with PBST, high-titer positive serum and blank negative serum were diluted in PBS to four different concentrations (1:100, 1:200, 1:400, 1:800) and incubated at 37°C for 1 h. Subsequently, 100 μL of HRP-conjugated Affinipure Goat Anti-Rabbit IgG was added to each well at four different concentrations (1:5000, 1:8000, 1:10000, and 1:12000) and incubated for four time periods (0.5 h, 1 h, 1.5 h, and 2 h). The plate was subsequently dried, washed three times with wash buffer, and 100 μL of single-component TMB was added to each well. The plate was incubated at 37°C in the dark for 15 min, after which 50 μL of stop solution (2 M H_2_SO_4_) was added. The absorbance at 450 nm was measured using a microplate reader.

### 2.8 Determination of the cutoff value

Under the optimal conditions established above, iELISA was performed on 30 BCoV positive bovine serum samples and 30 BCoV negative bovine serum samples, with each set of data replicated three times. The ideal cut-off value, as well as the diagnostic sensitivity and specificity, were established utilizing the Receiver Operating Characteristic (ROC) curve and Youden’s index (15).

### 2.9 Sensitivity and specificity evaluation of iELISA based on N protein

The sensitivity of the developed iELISA was evaluated by a two-fold serial dilution of high-titer rabbit pAb, spanning from 2^13^ to 2^19^, the maximum dilution factor at which iELISA successfully identified positive rabbit pAb was utilized to assess the assay’s sensitivity. A sample was deemed positive when the ratio of OD450 between positive (P) and negative (N) samples exceeded 2.1. To evaluate the specificity of the method, iELISA was employed to analyze positive serum samples for BCoV, BVDV, BRV, BRSV, and BHV-1, each serum sample was repeated three times.

### 2.10 Reproducibility analysis

The intra-assay and inter-assay reproducibility of iELISA was evaluated under the specified conditions applying three bovine positive serum samples and one bovine negative serum samples. Each sample underwent triplicate testing, and the mean, standard deviation, and coefficient of variation (CV) were computed for each sample to evaluate the stability and accuracy of the iELISA.

### 2.11 Comparison of iELISA and commercial iELISA kits

A total of 58 bovine serum samples were collected from cattle farms in Gansu Province and tested using the developed iELISA and a commercial antibody detection iELISA kit (Keshun Biotechnology, Shanghai, China). The results were compared to evaluate the relative sensitivity and specificity, and the concordance rate between the two methods was calculated. The concordance rate between the developed iELISA and the commercial kit results was determined by counting the number of consistent results between the two tests and dividing it by the total number of samples (16).

### 2.12 Statistical data analysis

Data processing and analysis were performed with GraphPad Prism 8 software. Each experiment had three independent replicates to ensure the reliability and statistical significance of the results.

## 3 Results

### 3.1 Sequence analysis and recombinant vector construction

**Figure 1:**
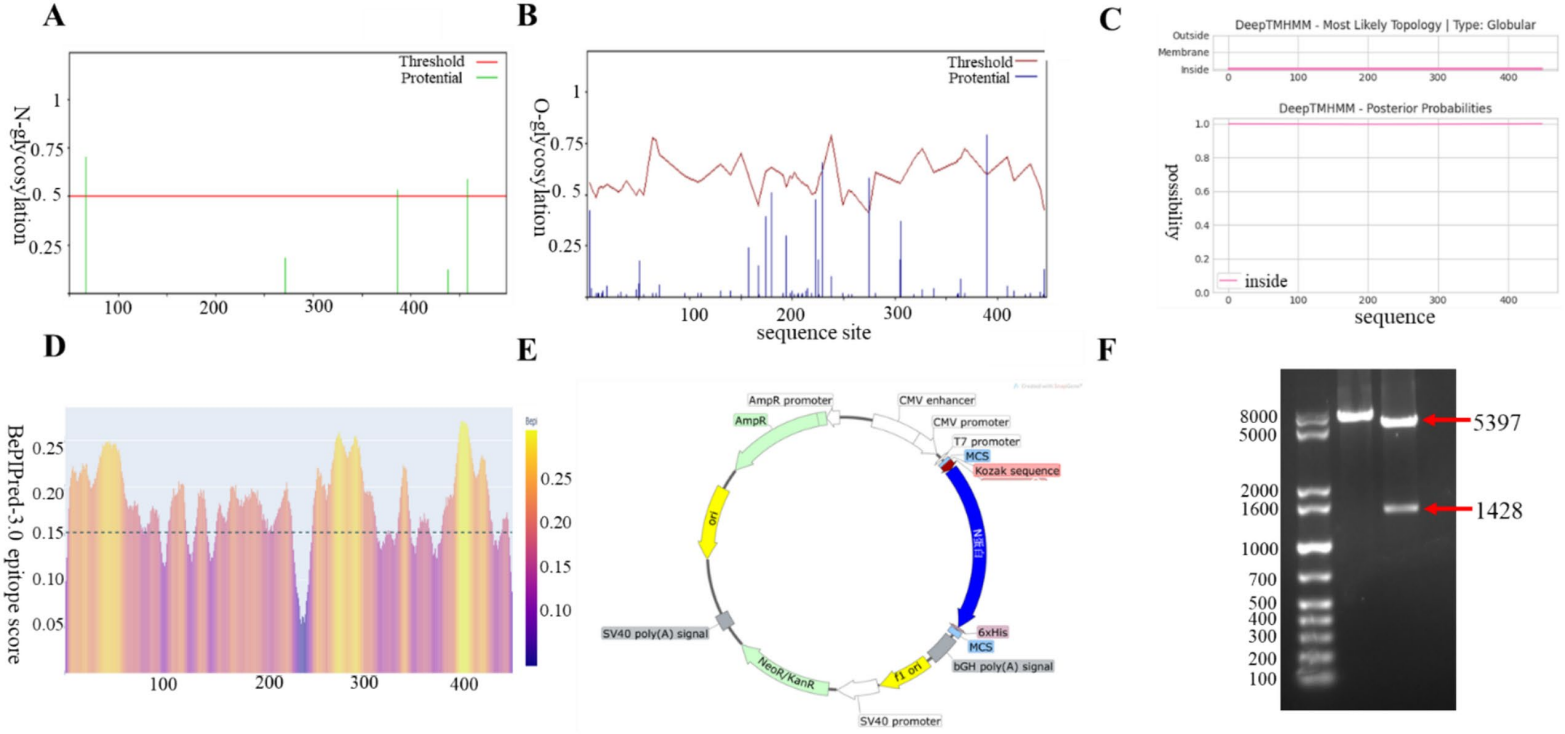
Construction and characterization of BCoV N protein eukaryotic expression vectors. **(A)** O-glycosylation analysis. **(B)** N-glycosylation analysis. **(C)** Signal peptide and transmembrane region prediction. **(D)** B-cell epitope prediction. **(E)** Construction mapping of pcDNA3.1-N prokaryotic expression vector. **(F)** Enzymatic characterization of pcDNA3.1-N prokaryotic expression vector. Lane M, 1 kb Plus DNA Marker; lane 1, *Eco*RI single digestion; lane 2, *Eco*RI and *Kpn*I double digestion.

The amino acid sequence of the N protein was predicted using an online bioinformatics tool. The analysis revealed the presence of three O-glycosylation sites and three N-glycosylation sites (Figures 1A,B), as well as the absence of a signal peptide and no structural domain of transmembrane region (Figure 1C). The N protein was found to be enriched with B-cell epitopes that exhibit strong immunogenicity, which is essential for antibody production (Figure 1D). The N sequence was codon optimized for CHO host cells, leading to an increase in GC content from 46.38 to 58.14%. It was subsequently cloned into the pcDNA3.1(+) vector, named as pcDNA3.1-N (Figure 1E). We identified the size of the *N* gene as 1,428 bp through double digestion, which was consistent with the theoretical value (Figure 1F). This further confirms the successful construction of the recombinant expression plasmid via DNA sequencing.

### 3.2 Recombinant N protein expression and purification identification

**Figure 2:**
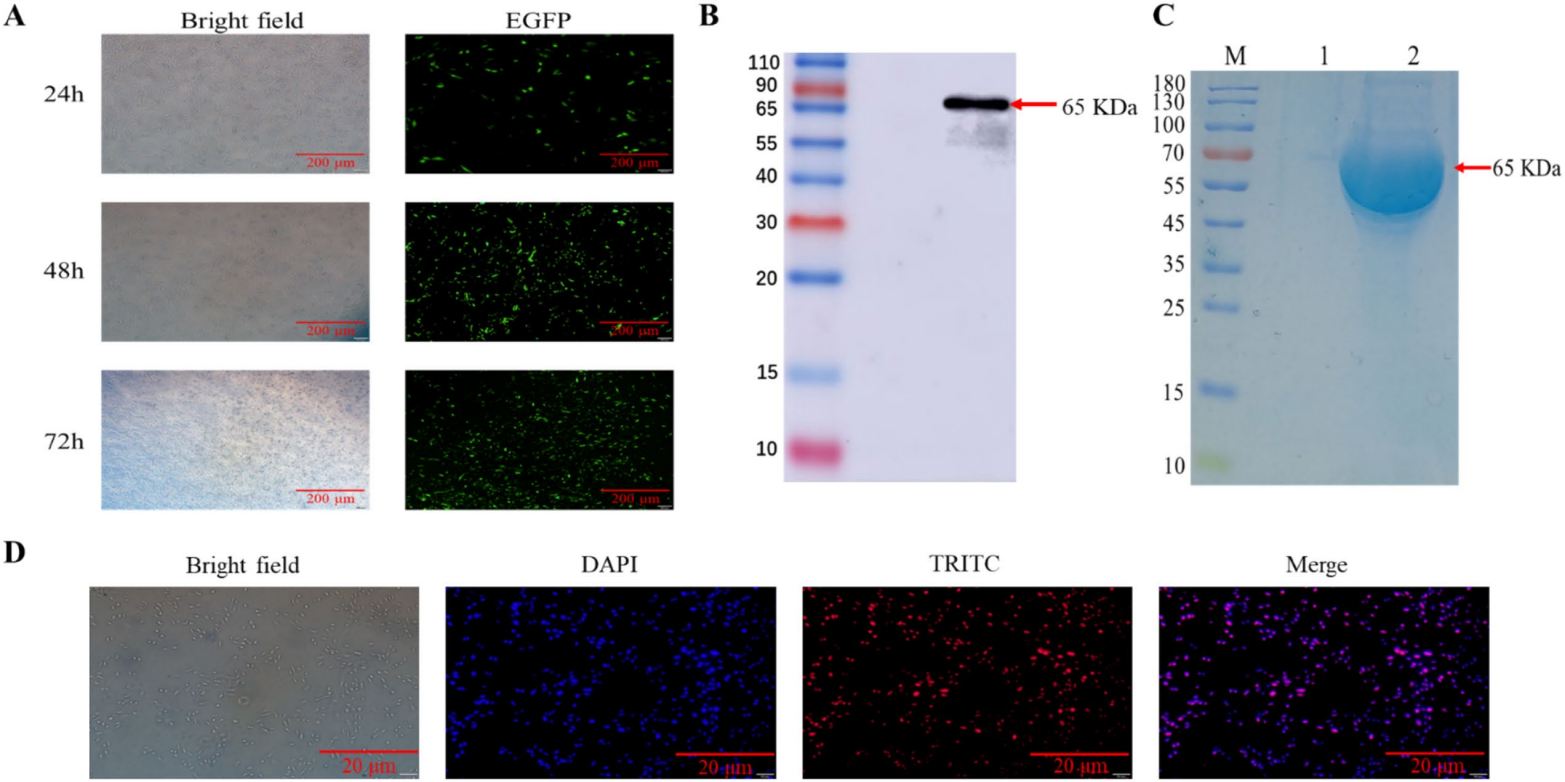
Eukaryotic expression and characterization of BCoV N proteins. **(A)** Protein expression analysis of eGFP transfected CHO-K1 cells. **(B)** Protein blotting identification of recombinant N protein using 6 × His rabbit monoclonal antibody, lane 1, supernatant of CHO-K1 cells; lane 2, supernatant of pcDNA3.1-N transfected CHO-K1 cells. **(C)** SDS-PAGE analysis of the recombinant N protein. Lane M, protein marker; lane 1, protein from untransfected cell supernatant; lane 2, purified N recombinant protein. **(D)** For IFA identification of BCoV-N protein, cells were fixed and immunostained with 6 × His monoclonal antibody followed by TRITC-conjugated Goat Anti-Rabbit IgG (H + L). Nuclei were restained with 4′,6-diamidino-2-phenylindole. The scale bar is 20 μm.

We characterized the transfection efficiency of the target gene by observing the expression of green fluorescent protein under fluorescence microscope, we collected CHO-K1 cells at three different time periods of 24, 48 and 36 h after transfection with eGFP, the results showed (Figure 2A) that eGFP protein levels increased with longer transfection times, with a significant rise at 48 h, peaking at 36 h. The results indicated that eGFP protein expression increased with prolonged transfection time, peaking at 36 h. we collected the supernatant of cell culture medium 3 days after transfection and purified it by affinity chromatography using Ni-NTA gravity column. WB and SDS-PAGE results indicated that the purified N protein exhibited a single band at 65 kDa, which exceeds the anticipated size of 53 kDa (Figures 2B,C). This discrepancy may be attributed to post-translational modifications of the recombinant N protein in eukaryotic cells. Subsequent IFA experiments were conducted to further validate the expression of the N protein in CHO-K1 cells (Figure 2D).

### 3.3 Preparation and characterization of rabbit pAb

We used recombinant N protein as the coated antigen to determine the potency of rabbit pAb serum as 8.192 × 10^5^ (Table 1).

**Table 1:** Rabbit antiserum potency of BCoV N protein.

Serum dilution (×100)	Positive (P) OD450	Negative (N) OD450	P/N
1	3.97	0.30	13.25
2	3.66	0.29	12.63
4	4.12	0.28	14.70
8	3.83	0.28	13.69
16	3.40	0.21	16.20
32	3.74	0.27	13.84
64	3.86	0.22	17.57
128	3.30	0.21	15.70
256	3.31	0.22	15.07
512	2.65	0.19	13.93
1,024	0.73	0.17	4.31
2048	0.72	0.14	5.17
4,096	0.59	0.13	4.54
8,192	0.31	0.14	**2.20**
16,384	0.25	0.16	1.56

**Figure 3:**
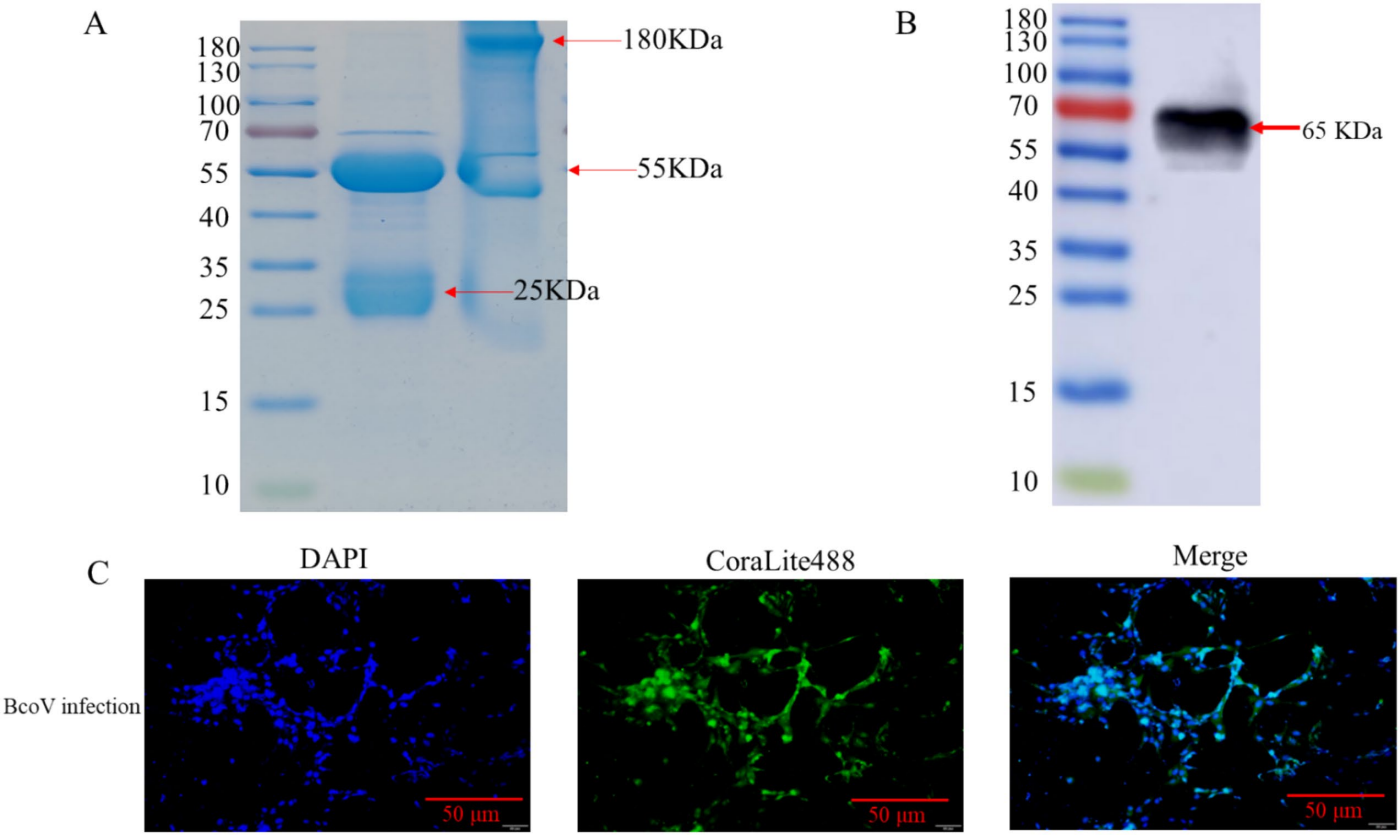
Characterization and identification of rabbit pAb against N protein. **(A)** SDS-PAGE analysis of purified pAb. Lane M, standard protein marker; lane 1, purified non-reduced pAb; lane 2, purified reduced pAb. **(B)** Protein blotting identification of recombinant BCoV N protein using purified antiserum as primary antibody; lane M, protein marker; lane 1, recombinant N protein. **(C)** IFA analysis of pAb against BCoV N protein. MDBK cells were infected using BCoV, cells were fixed 48 h after infection and stained with purified rabbit pAb and CoraLite488-conjugated Goat Anti-Rabbit IgG (H + L), Cell nuclei were counterstained with 4′,6-diamidino-2-phenylindole at a scale of 50 μm.

Following the purification of the obtained rabbit serum by affinity chromatography, the structures of reduced and non-reduced rabbit pAb were characterized (Figure 3A). The molecular weights of non-reduced antibodies were approximately 180 KDa, consistent with expectations and maintaining their natural structure, while the molecular weights of reduced antibodies were approximately 55 KDa and 25 KDa, respectively. The WB results demonstrated that the purified rabbit pAb exhibited strong immunoreactivity with the recombinant N protein (Figure 3B). We employed BCoV to infect MDBK cells, and the IFA results showed that the rabbit poly antibody specifically recognized BCoV. These results demonstrated that the rabbit pAb exhibited strong immunoreactivity and specificity toward both recombinant N protein and BCoV, and that it was promising to be developed as a pathogen detection reagent (Figure 3C).

### 3.4 Optimization of conditions for iELISA based on N protein

**Figure 4:**
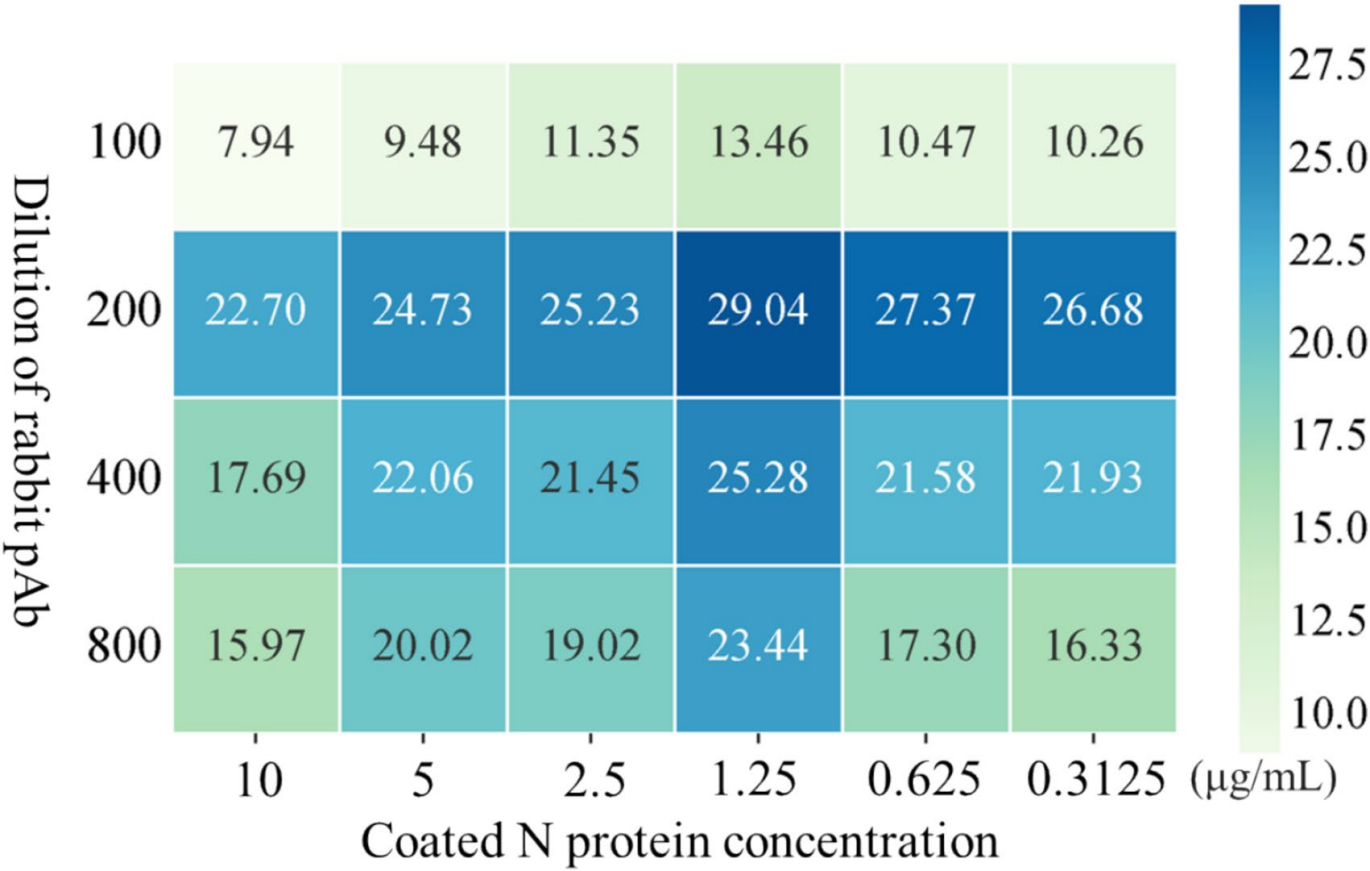
Optimization of iELISA method based on N protein. The optimal working concentrations of coated N antigen and rabbit pAb were determined using checkerboard titration. The magnitude of the OD450 value is shown in the heatmap, darker colors indicate higher OD450.

The conditions of iELISA were optimized based on the purified N protein, the results of the checkerboard titration showed that the optimal antigen concentration was 1.25 μg/mL, and the optimal dilution of the antiserum is 200-fold, yielding a P/N value of 29.04 (Figure 4).

**Figure 5:**
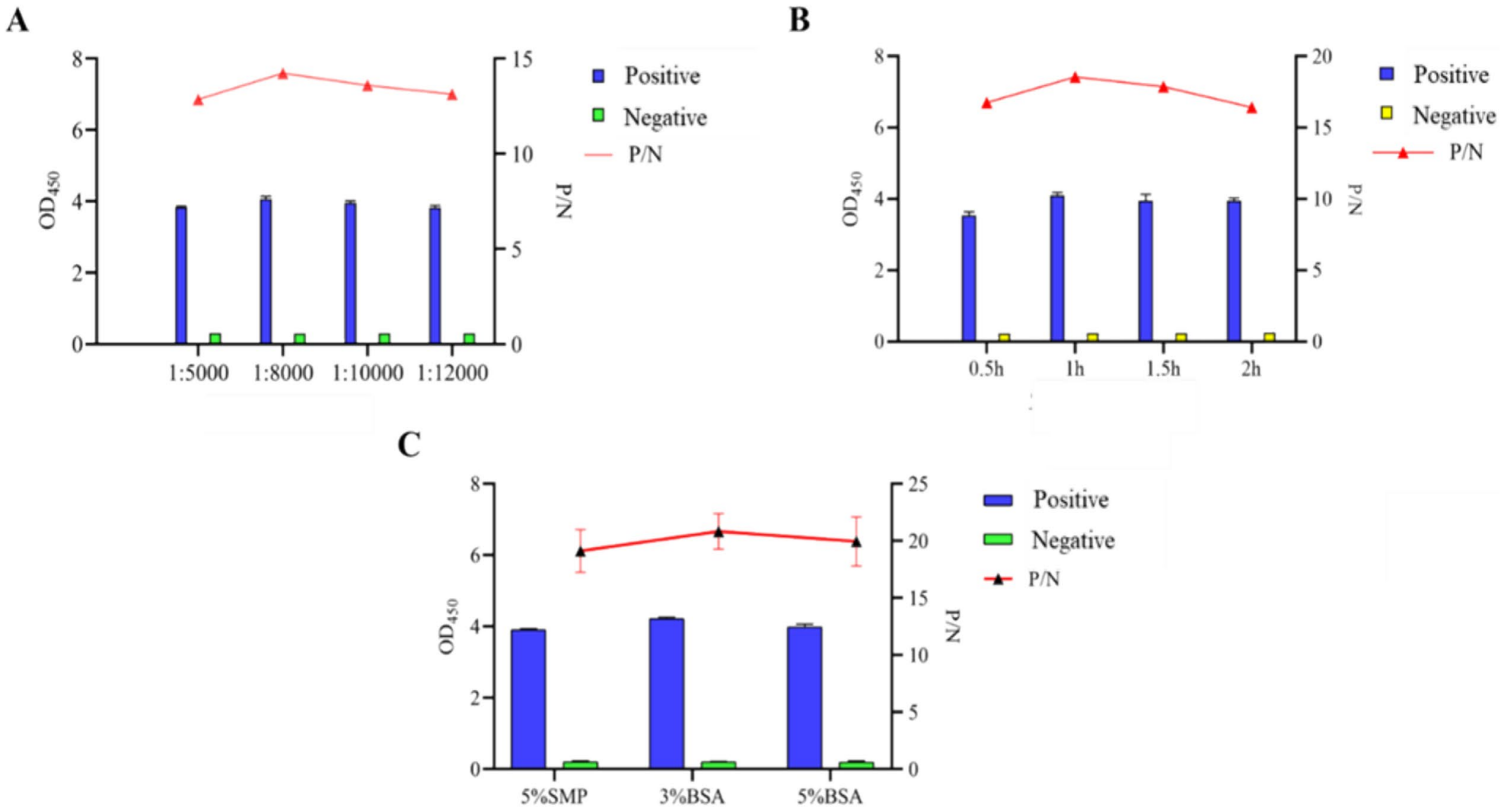
Optimization of indirect ELISA conditions based on N protein. **(A)** Optimization of working dilution of HRP-conjugated Affinipure Goat Anti-Rabbit IgG secondary antibody. **(B)** Optimal incubation time of recombinant gD protein coupled with HRP-coupled secondary antibody. **(C)** Determination of the types of blocking agents.

Subsequently, other important conditions were also improved, and the experimental results showed that the optimal results were obtained when the blocking agent was 3% BSA, and the dilution of the secondary antibody was 1:8,000 and the incubation time of secondary antibody was 1 h, the best reaction result was achieved (Figure 5).

### 3.5 Optimal cutoff, diagnostic specificity and sensitivity determination

**Figure 6:**
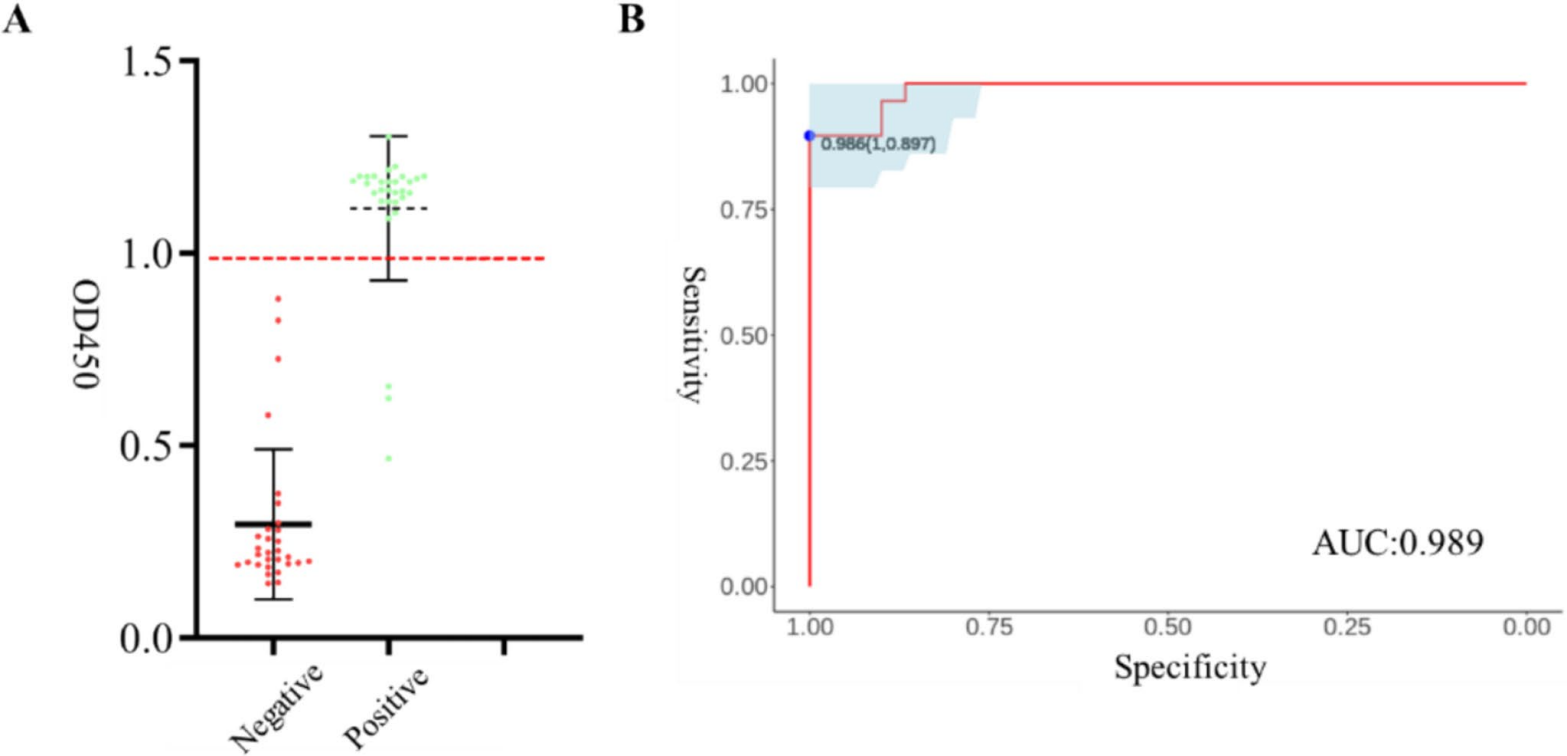
Analysis of critical value, assay specificity, and sensitivity of indirect ELISA based on N protein. ROC analysis using 60 known positive and positive clinical samples. **(A)** OD_450_ of 60 clinical samples with red dashed line as critical value. **(B)** ROC curve to determine critical value, specificity and sensitivity and AUC.

We employed iELISA to analyze 30 BCoV positive serum samples and 30 negative serum samples for data quantification (Figure 6B), and determined the critical value of the method by producing a subject operating characteristic curve (ROC). The ROC analysis indicated that the diagnostic sensitivity and specificity of iELISA were 89.7 and 100%, respectively, with an optimal cutoff value of 0.986, and a Youden index of 0.897 (Figure 6A). The proximity of the area under the curve (AUC) of the ROC to 1.0 is widely regarded as indicative of assay authenticity. The findings of the iELISA demonstrated an AUC value of 0.989 (95% CI = 0.971 ~ 1.000), signifying that the method exhibits high precision. A sample was classified as “positive” if the measured OD450 value was >0.986, whereas a sample with an OD450 value <0.986 was classified as “negative.”

### 3.6 Sensitivity determination of iELISA

**Figure 7:**
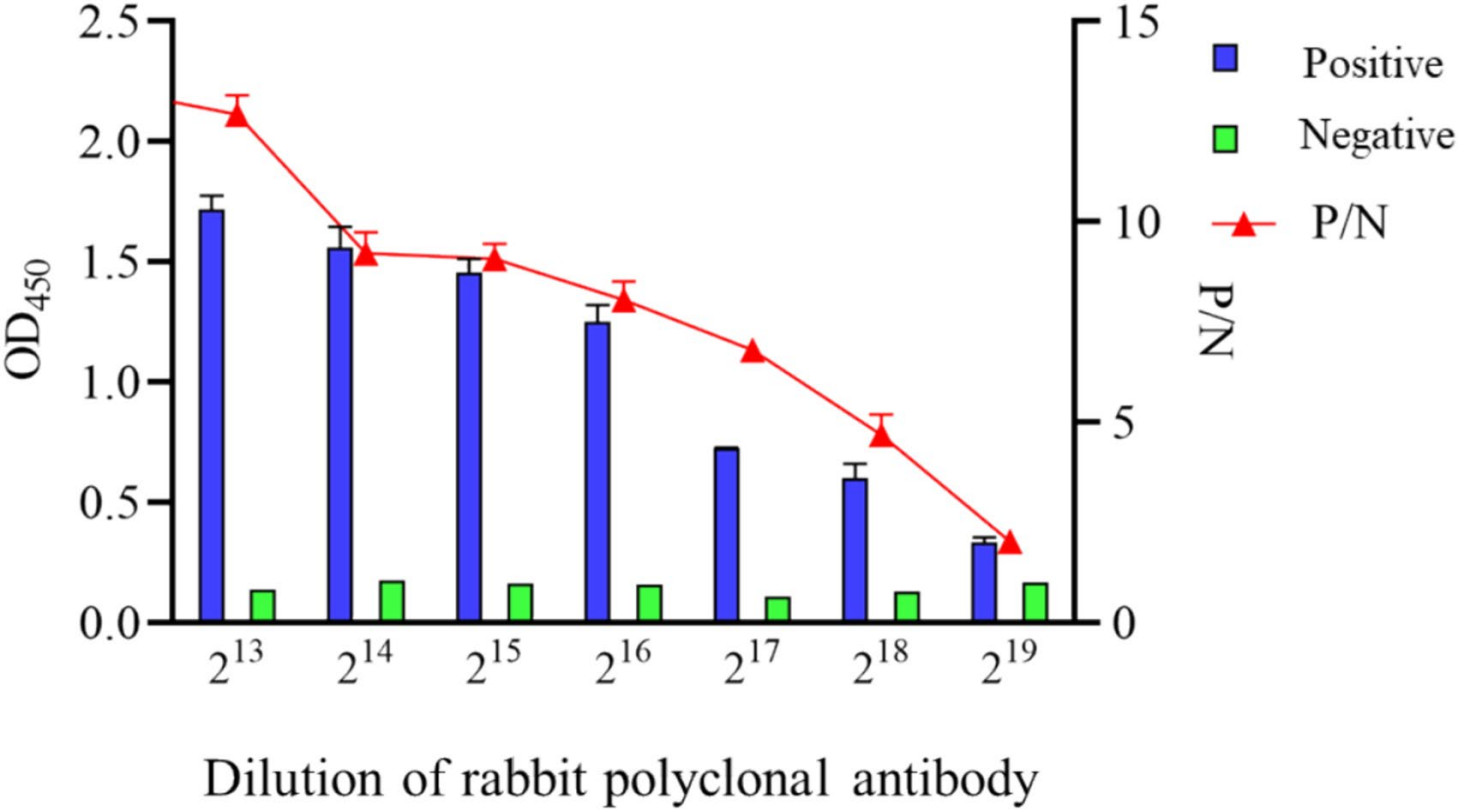
Sensitivity analysis of indirect ELISA based on N protein.

To ascertain the detection limit of the iELISA based on N protein, the rabbit pAb was diluted 2-fold and analyzed by the established iELISA method. The results showed a P/N value of 1.986 at a dilution of 2^19^, thus the maximum dilution at 2^18^ (Figure 7).

### 3.7 Assessment of specificity of iELISA

**Figure 8:**
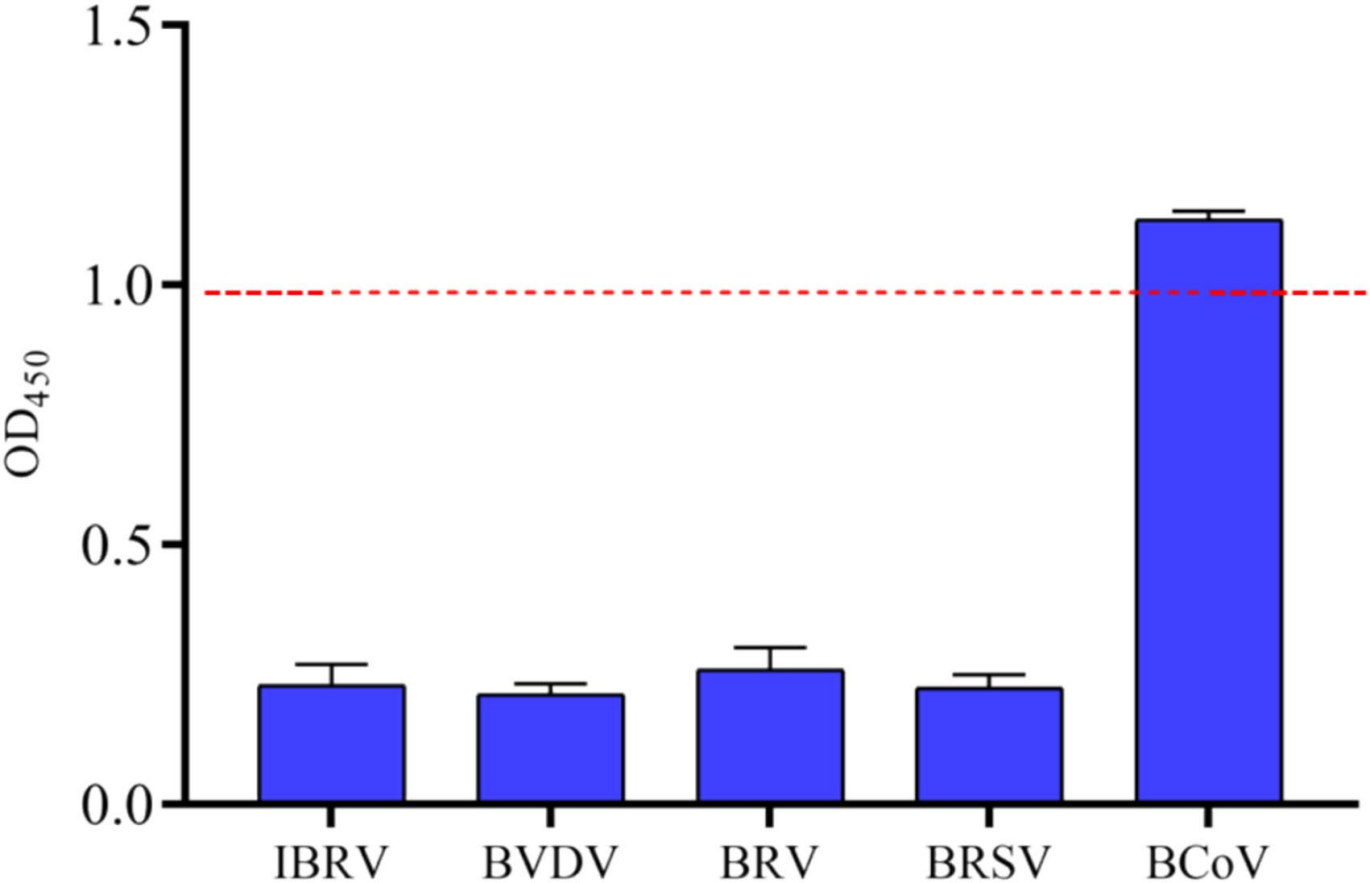
Specificity analysis of indirect ELISA based on N protein. Based on the method established above, OD_450_ values of clinical samples of BCoV, BVDV, BRV, BRSV, BHV-1 were tested, and the critical values for negatives and positives are indicated by dashed lines.

To evaluate the specificity of iELISA, cross-reactivity tests were performed using bovine positive serum samples of common bovine viruses, including BHV-1, BVDV, BRV, BRSV, and BCoV. The results showed that, except for the BCoV serum samples, the OD450 values of other serum samples were lower than 0.986 with significant differences in values, while the positive serum containing BCoV showed strong reactivity with an OD450 value of 1.125 (Figure 8). The results indicated that the iELISA exhibited no cross-reactivity with other prevalent bovine virus sera and had strong specificity for BCoV sera.

### 3.8 Repeatability analysis

Repeatability was tested using three bovine positive serum samples and one bovine negative serum sample. The results in the table showed that the CV ranged from 1.661 to 3.773% within batches and from 0.291 to 3.381% between batches, which were not more than 5%, leading to good repeatability (Table 2).

**Table 2:** Repeatability analysis of the indirect ELISA method based on N protein.

Serum samples		1	2	3	Average	Standard deviation	CV (%)
P1	Intra-batch	1.200	1.130	1.211	1.180	0.044	3.722
P2		1.188	1.227	1.216	1.210	0.020	1.661
P3		1.162	1.145	1.182	1.163	0.019	1.592
N		0.218	0.214	0.230	0.221	0.008	3.773
P1	Inter-batch	1.135	1.200	1.130	1.155	0.039	3.381
P2		1.122	1.123	1.155	1.133	0.019	1.656
P3		1.044	1.037	1.085	1.055	0.026	2.457
N		0.198	0.198	0.199	0.198	0.001	0.291

### 3.9 Comparison between iELISA and the commercial kit

Fifty-eight bovine serum samples collected from Gansu Province, China, were tested using a commercial kit and compared with the developed EILSA method. Five BCV-positive samples were detected by the commercial kit, whereas eight positive samples were detected by the developed iELISA method. The diagnostic sensitivity and specificity of the developed ELISA method were 100 and 94.64%, respectively, and the concordance rate between the two methods was 94.83% (Table 3), indicating that the method is suitable for the rapid diagnosis of clinical samples.

**Table 3:** Tests of clinical serum samples.

Serum samples	Commercial ELISA	Development ELISA	Sensitivity	Specificity	Coincidence rate
Positive	5	8	100% (5/5)	94.64% (53/56)	94.83% (55/58)
Negative	53	50			

## 4 Discussion

Dairy and beef cattle production plays an important role in the livestock industry, with the quality of cattle-derived products directly impacting human food health. BCoV is a significant pathogen responsible for diarrhea in neonatal calves and adult cattle, resulting in considerable economic losses for the cattle industry. BCoV exhibits a global distribution, with positivity rates differing substantially based on geographic location, rearing practices, farm size, and herd age (17). In Heilongjiang Province, China, the BCoV positivity rate was recorded at 15.45% (157/1016) through reverse transcription polymerase chain reaction (RT-PCR) (18). A meta-analysis in China (19) showed that the overall prevalence of BCoV was 30.8%, with the highest prevalence observed in South China (60.5%) and the lowest in Central China (15.6%). Sixteen out of 140 fecal samples from calves with diarrhea, collected in Korea (20) between 2017 and 2018, tested positive for BCoV. Specific antibodies against BCoV were detected in 72.6% (215/296) of animals on cattle farms in Poland via RT-PCR (21). Moreover, BCoV belongs to group 2a coronaviruses along with SARS-CoV-2, which is the most prevalent cause of severe respiratory diseases in humans currently, its extensive host range and elevated incidence of genomic recombination events pose a great potential threat to human health. The current lack of safe commercial vaccines and effective therapies for prevention and treatment underscores the importance of developing rapid and accurate diagnostic methods to detect BCoV infection.

BCoV N protein is a highly conserved structural protein in BCoV and is considered a promising diagnostic marker. Presently, most people often use RT-PCR based on *N* gene primers for epidemiological studies of BCoV, which is a classical and reliable method for molecular level detection (22–24). However, it is hindered by a laborious operational process, extended experimental duration, and costly equipment, resulting in an enormous consumption of both material and financial resources in large-scale clinical sample detection. Currently, ELISA is considered by the World Organization for Animal Health as one of the most commonly used methods for animal pathogen detection, with high sensitivity and accuracy. Furthermore, it facilitates batch testing at a cheap cost, becoming it the most favored approach currently (25, 26). Consequently, we have developed an iELISA method based on N protein, a diagnostic method whose efficacy primarily relies on the binding affinity between the antigen and the antibody, as well as the quality of the antibody and its interaction with the antigen. In this study, we used a CHO expression system for the secretory expression of the protein, so that the expressed N protein can be folded correctly to ensure the original spatial conformation and protein activity. This approach aims to closely resemble the native protein, thereby enhancing its immunogenicity during immunization. The expression level and purity of N protein are crucial to the performance of the iELISA method. CHO cells are a cell line that can grow both adherently and in suspension. They have the ability to efficiently amplify and express recombinant genes and the function of product extracellular secretion (27). They are currently the preferred system for recombinant protein production and are widely used in the research and development and production of products such as antibodies (28), recombinant protein drugs (29) and vaccines (30). Many marketed protein drugs are produced based on CHO cells, such as tissue plasminogen activator (31), erythropoietin (32), rituximab (33), etc. Previous studies have shown that through an optimized perfusion culture strategy, CHO cells were cultured in a 200 L bioreactor, and the final product titer reached the highest level of 16.79 g/L on the 16th day (34). The disadvantage is that we use adherent cells for protein expression, which results in a cumbersome purification process and low expression level. This is because the growth density of adherent cells is usually low, and serum needs to be added during culture, the serum components are complex and contain a variety of impurities (35). These impurities will mix with the target protein, increasing the difficulty of purification (36). Suspension cells have the advantages of simple culture medium components, high cell density and easy large-scale culture (37, 38). This requires the use of suspension cells in subsequent studies to improve the high-level expression and purification efficiency of target protein.

The primary diagnostic methods for BCoV identified include RT-PCR, multiplexed real-time fluorescence quantitative PCR, and a combination of isothermal rapid amplification with multiple enzymes and lateral chromatography paper (39). While these methods exhibit high accuracy, they are complex and time-consuming, rendering them unsuitable for the detection of a broad spectrum of clinical samples. ELISA serves as a rapid, simple and sensitive method for the detection of animal pathogens, but there are few reports on ELISA assays for BCoV. Researchers in Norway used a multiplex immunoassay for BCoV with a commercial iELISA (SVANOVIR BCV-Ab) to detect antibodies in bulk milk tank samples. The multiplex assay used a set of three recombinant proteins (A-C) as antigens, and the commercial iELISA used an unknown BCoV antigen to detect antibodies (IgG) in bulk milk samples. Under optimized conditions, using a Bayesian latent class model, the researchers found that the sensitivity of the multiplex assay was 99.9% and the specificity was 93.7% (40). We developed an iELISA based on N recombinant protein, optimizing the reaction conditions to achieve an optimal critical value of 0.986 as determined by the ROC curve. This value was significantly different from that of negative samples, with a Youden’s index of 0.897, indicating good model performance. The diagnostic sensitivity and specificity of the established ELISA method for testing 58 serum samples were 100 and 94.64%, respectively, and the concordance rate between the developed ELISA method and the commercial kit was 94.83%. Although we tested serum samples instead of milk samples, such results are surprising, indicating that the iELISA method has the potential to be widely used in BCoV herd surveillance and control programs. Although the results are good, the study only included a limited number of clinical samples for testing and validation, and milk samples should also be tested to understand other factors that may affect the performance of the test and the relationship between sample type and test method (41).

In the sensitivity experiment, the maximum dilution of the antiserum was 2^18^, which had a very high detection limit, which was similar to the detection limit of the iELISA method based on the VP6 protein expressed in eukaryotic cells for the detection of bovine rotavirus (42). This may be due to the complete glycosylation modification and natural conformation of the N protein expressed in the eukaryotic system (43). The establishment of this method can simplify the process and steps of BCoV detection in large-scale cattle farms, greatly reduce the loss of manpower and material resources, improve detection efficiency, and provide a powerful tool for the prevention and control of BCoV.

## 5 Conclusion

In conclusion, we effectively expressed the secreted recombinant N protein utilizing CHO-K1 cells, subsequently employing it as the immunogen to generate rabbit pAb. We developed an iELISA method based on N protein for the detection of BCoV antibodies in clinical serum samples, demonstrating high sensitivity and specificity, with no cross-reactivity with other bovine-associated virus sera, and exhibiting strong reproducibility. The results indicate that the iELISA developed in this study serves as a dependable detection tool for the prevention and control of BCoV transmission.

## Data Availability

The raw data supporting the conclusions of this article will be made available by the authors, without undue reservation.
